# Impact of a mindfulness-based cognitive behavioral group intervention on psychological outcomes in women with endometriosis: a controlled clinical study plan and protocol

**DOI:** 10.1192/j.eurpsy.2026.11720

**Published:** 2026-07-31

**Authors:** Á. Zinner-Gérecz, X. Gonda, S. Kresznerits

**Affiliations:** Department of Clinical Psychology, Semmelweis University, Budapest, Hungary

## Abstract

**Introduction:**

Endometriosis is a chronic, systemic, estrogen-dependent gynecological disorder affecting approximately 10% of women of reproductive age. Beyond the hallmark physical symptoms, the condition is associated with significant psychological burden. The disease not only generates local inflammation but also affects central pain modulation and gene expression in brain regions responsible for emotional regulation.

**Objectives:**

Our current study aims to evaluate the feasibility, applicability, and effectiveness of an 8-session mindfulness-based cognitive behavioral group intervention for women diagnosed with endometriosis. The primary focus is to examine changes in psychological functioning, mental wellbeing, sexual function, perceived pain, and health-related quality of life, as well as to identify psychological predictors of therapeutic response, with particular attention to affective temperament profiles.

**Methods:**

The intervention will be delivered in a non-randomized, waitlist-controlled design (N = 80–100). Participants will be recruited through gynecological outpatient services, following a verified diagnosis of endometriosis by board certified gynecologists. The group therapy combines mindfulness practices (e.g., body scan, loving-kindness, breath-focused awareness) with classical cognitive behavioral techniques (e.g., cognitive restructuring, thought monitoring) and psychoeducational content. Psychological assessments will be conducted pre-intervention, post-intervention, and at 3- and 6-month follow-up visits. Instruments include the Patient Health Questionnaire-9 (PHQ-9), State-Trait Anxiety Inventory (STAI), Short Form Health Survey (SF-36), Temperament Evaluation of Memphis, Pisa, Paris and San Diego (TEMPS-A), Schema Mode Inventory (SMI), a numerical pain rating scale, and the Female Sexual Function Index (FSFI).

**Results:**

We hypothesize significant improvements in psychological and functional outcomes in the intervention group compared to controls, including reductions in depression and anxiety scores (p < .05), decreased perceived pain, and enhanced quality of life and sexual functioning. Furthermore, we expect that specific temperament traits, along with demographic and baseline clinical factors, may predict the degree of therapeutic benefit.

**Conclusions:**

The findings are expected to provide strong preliminary evidence for the integration of structured psychological interventions into the multidisciplinary care of endometriosis. Given the current lack of such programs in Hungary, this study may contribute to developing a sustainable, patient-centered treatment model that addresses both physical and psychological dimensions of the disease.

**Disclosure of Interest:**

None Declared

